# Interactive editing of virtual chordae tendineae for the simulation of the mitral valve in a decision support system

**DOI:** 10.1007/s11548-020-02230-y

**Published:** 2020-10-24

**Authors:** Lars Walczak, Lennart Tautz, Mathias Neugebauer, Joachim Georgii, Isaac Wamala, Simon Sündermann, Volkmar Falk, Anja Hennemuth

**Affiliations:** 1 Fraunhofer MEVIS, Bremen, Germany; 2 Charité—Universitätsmedizin Berlin, Berlin, Germany; 3 German Heart Institute Berlin—DHZB, Berlin, Germany; 4 German Centre for Cardiovascular Research—DZHK, Berlin, Germany; 5 Swiss Federal Institute of Technology, Zürich, Switzerland

**Keywords:** Decision support, Mitral valve, Position-based dynamics

## Abstract

**Purpose:**

Decision support systems for mitral valve disease are an important step toward personalized surgery planning. A simulation of the mitral valve apparatus is required for decision support. Building a model of the chordae tendineae is an essential component of a mitral valve simulation. Due to image quality and artifacts, the chordae tendineae cannot be reliably detected in medical imaging.

**Methods:**

Using the position-based dynamics framework, we are able to realistically simulate the opening and closing of the mitral valve. Here, we present a heuristic method for building an initial chordae model needed for a successful simulation. In addition to the heuristic, we present an interactive editor to refine the chordae model and to further improve pathology reproduction as well as geometric approximation of the closed valve.

**Results:**

For evaluation, five mitral valves were reconstructed based on image sequences of patients scheduled for mitral valve surgery. We evaluated the approximation of the closed valves using either just the heuristic chordae model or a manually refined model. Using the manually refined models, prolapse was correctly reproduced in four of the five cases compared to two of the five cases when using the heuristic. In addition, using the editor improved the approximation in four cases.

**Conclusions:**

Our approach is suitable to create realistically parameterized mitral valve apparatus reconstructions for the simulation of normally and abnormally closing valves in a decision support system.

## Introduction

Mitral valve (MV) regurgitation (MR) is the second most common heart valve disease with an increasing incidence [6]. Roughly 12,300 MV procedures were performed in Germany in 2017 [1]. Patients with MR exhibit defects in the leaflets, annulus, or chordae tendineae (CT, tendinous cords), but other pathological alterations are possible as well. The result is an incomplete closure or a prolapsing of the leaflets into the left atrium (LA), which, in turn, leads to blood flow back into the LA during heart contraction. Heart function is impaired and volume load on the LA as well as the lungs is increased. The gold standard therapy for MR is surgical reconstruction [13, 14]. To further improve the planning process prior to an intervention and to find an optimal combination of treatments, a truly personalized planning process implemented in a decision support system is needed.

**Fig. 1:**
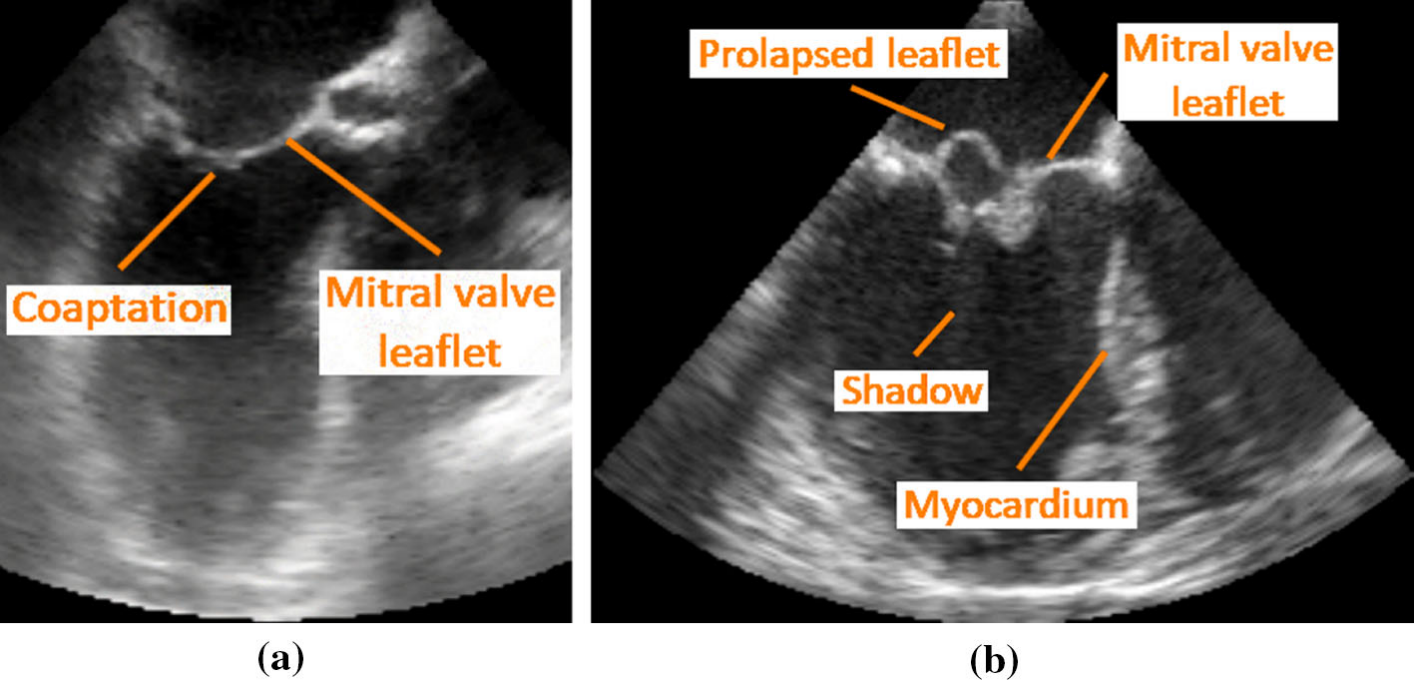
**a–b** Long axis views of the left heart. Some anatomical structures, landmarks, and acquisition artifacts are highlighted. Disadvantages of TEE acquisitions are shadowing and signal dropout artifacts. In addition, the leaflets can be positioned very close to the myocardium in the open state, while in the closed state, the coaptation zone (area where the leaflets meet) can be difficult to assess. **a** Normally closing valve; **b** abnormally closing valve showing prolapse

To support therapy planning with quantifiable numbers and visualizations, simulation systems such as the ones discussed in [3, 16] have to be included in decision support systems. Using said systems, virtual interventions modifying reconstructed patient-specific geometry can be safely explored, compared, analyzed, and visualized. But since the setup of such simulations using patient-specific MV geometry is based on clinical imaging, it involves a number of uncertainties. Especially imaging artifacts and image quality play critical roles for the reconstruction of the patient-specific state. In the following, we address one specific aspect in the simulation pipeline, namely the modeling of chordae tendineae. Chordae tendineae connect the MV leaflets to the muscles located in the wall of the heart. These tendons are tiny and cannot be reliably seen in most commonly used imaging techniques for MV interventions, e.g., echocardiography [3, 16]. For truly personalized medicine using virtual operation simulation systems, the cords have to be modeled in order to reproduce the current state. This represents the base state for exploration and enables modifications. In addition to a heuristic model for the initial approximation of the chordae tendineae, we describe an interactive editor for fine-tuning the cord rest lengths as well as the papillary muscle tip locations. Using this technique, we are able to optimize the geometric approximation as well as improve pathology reproduction. We are not aware of other solutions for interactively editing and simulating the MV apparatus based on patient-specific image data.

Medical background The MV is part of the left heart (LH) and is positioned between the LA and the left ventricle (LV). The MV’s two leaflets are attached to the mitral annulus (MA) and the CT. The CT themselves are attached to the papillary muscles (PM), which extend from the wall of the heart into the ventricle. The MV is open during the ventricle filling phase in which oxygenated blood coming from the lungs fills the LV by passing through the LA. When fresh blood is ejected into the aorta in the contraction phase, the MV is closed. Blood flow back to the LA is normally prevented by a combined parachute-like action of leaflets, chordae tendineae, and papillary muscles. Cords and papillary muscles keep the leaflets in place during systole and prevent them from prolapsing into the atrium. Therefore, the chordae tendineae are an important component of the mitral apparatus. This process is called coaptation. An improper coaptation, e.g., caused by ruptured chordae tendineae, leads to MR, cf. Fig. 1.

**Fig. 2:**
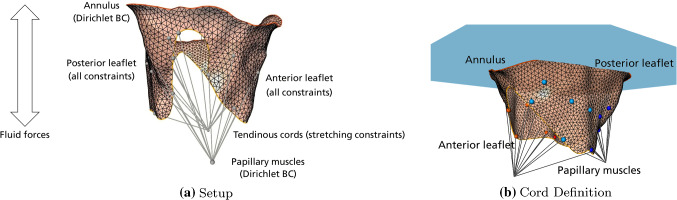
**a** Overview of the complete simulation setup. **BC** refers to boundary condition. **b** Setup for defining the chordae tendineae. The papillary muscle tips $\mathcal {V}^{PM}_{i}$ are connected to the leaflet vertices $\mathcal {V}_{j}$ via edge $\mathcal {E}^{CT}_{ij}$ (gray lines). In this case, light blue and light red colored vertices connect to one PM tip, while the darker blue and darker red colored vertices are connected to the other PM tip. The annulus plane (AP) is shown in blue. The AP is used for initial length determination of all $\mathcal {E}^{CT}_{ij}$ (see text)

Current basis for therapy decisions in MR is the visual as well as the quantitative assessment of the patient’s valve anatomy and function through imaging. The gold standard diagnostic tool is echocardiography. Transesophageal echocardiograms (TEE) are acquired before, during, and after MV intervention. Although exhibiting artifacts, such as signal dropout or shadowing effects (cf. Fig. 1b), the images are used for planning, guiding, and success rating. A relatively high temporal and spatial resolution of the 3D $+$ t image data sets enables measurements and visualization of the MV in different cardiac phases. Although the data can be used for reconstructing the MV, e.g., by using an approach similar to [15], the data cannot be used for reconstructing the patient specific CT configuration. The scale of these structures is simply too small, and their exact location can only be estimated.

After image analysis, the further planning of the intervention has to be discussed and prepared in the surgeons’ minds. A decision support system (DSS) for MV interventions including modules for reconstructing and simulating the patient-specific MV opening and closing behavior under normal and altered conditions can be used for visual analyses and choice of therapy. The simulation system described in [16] provides interactive modules for the exploration of different intervention alternatives and thus provides a visualization of the patient’s pre- and virtual post-operative state. The alternatives can be safely explored in silico. After a thorough analysis of the outcome, the results of the simulation after virtual intervention may provide new insights into the modified dynamics and, in turn, may influence the therapy decision [9].

## Methods

Since we concentrate on describing an interactive editor for defining and optimizing the cord model, we only briefly summarize the simulation model used here and refer the reader to [16] for more in-depth information.

### Mitral valve model and simulation

The simulation model follows the approach presented in [16] and can be executed at interactive frame rates on current PC hardware. The simulation itself is implemented using the MeVisLab platform [12].

In [16], the MV shape is approximated by a triangular mesh which is created semi-automatically based on the patient’s image data showing the open state MV. In this state, both leaflet ends are separated from each other and can most likely be correctly identified and differentiated. In addition to the open state valve, the annulus contour for the closed state has to be defined. A more detailed description of the segmentation approach used here can be found in [15].

The resulting mesh approximation of the MV consists of vertices $\mathcal {V}_i$, edges $\mathcal {E}_{ij} = (\mathcal {V}_i, \mathcal {V}_j), i \ne j$, and triangles $\mathcal {T}_{ijk} = (\mathcal {V}_i, \mathcal {V}_j, \mathcal {V}_k), i \ne j \ne k$. The initial open state is referred to as time $t=0$. The reconstructed mesh is augmented with a set of constraints modeling the valve material, see Fig. 2a. The extended positions-based dynamics (XPBD, [7]) approach taken in [16] uses linearly elastic distance constraints, bending constraints as well as area conservation constraints to model the MV leaflet material. The constraints are set up using the geometric relations of the mesh at $t=0$. Each edge $\mathcal {E}_{ij}$ of the mesh is converted into a distance constraint. Distance constraints model springs, and their rest lengths $l_0$ are set to the initial distances between the two vertices $\mathcal {V}_i, \mathcal {V}_j$ incident to the edge. Bending constraints are created between neighboring triangles $\mathcal {T}_{ijk}, \mathcal {T}_{ijl}, k \ne l$. They are used to restrict the dihedral angle between both triangles, e.g., to the initial angle between both faces. In addition, an area constraint is generated for each triangle $\mathcal {T}_{ijk}$. By adding area constraints, the initial total surface area of the valve is conserved during simulation. Contact is modeled using collision constraints which allow the two leaflets to coaptate or glide off each other because of the driving external forces (details in [16]).

The external forces model the blood flow in a simplified manner and consist of different pressure and velocity fields for representing systole and diastole, see Fig. 2a. The pressure force is defined using the normal $\mathbf {n}_i$ at vertex $\mathcal {V}_i$ while the main component of the velocity field is the flow direction through the valve. The main flow direction is assumed to correspond to the annulus plane (AP) normal $\mathbf {n}^{AP}$. The AP is found, e.g., using principle component analysis of the annulus contour. Using Newton’s second law of motion and numerical integration, the valve moves according to the fields. The valve can be dynamically closed and opened by changing the signs of the fields. To account for the properties of the modeled material, a separate nonlinear Gauss–Seidel solver that satisfies the constraints is needed (details in [16]). The annulus contour is represented by a Dirichlet boundary condition in the simulation and is interpolated between open (at $t=0$) and closed ($t>0$) state, see Fig. 2a.

### Modeling of tendinous cords

Using the model above, both leaflets are fixed at the annulus. The loose leaflet ends at the orifice as well as the parts in between orifice and annulus are able to move based on the material model and the forces applied. To model the actual physiologic behavior and thus complete the MV model in [16], a definition of the tendinous cords is needed to model valve closure. Because the simulation starts with the valve being in an open state at time $t=0$, we define the cords in this state.

Previous results [4, 5] indicate that an exact cord model may not be needed for modeling valve closure. Following these results, we simplify and approximate the whole cord tree by a set of links that we model as additional edges $\mathcal {E}^{CT}_{ij} = (\mathcal {V}^{PM}_{i}, \mathcal {V}_{j})$ in the simulation. These edges can be defined between either manually or heuristically defined papillary muscle tip vertices $\mathcal {V}^{PM}_{i}$ and manually defined leaflet vertices $\mathcal {V}_{j}$ (cf. Fig. 2b). For a manual definition of both types of end points, we use MeVisLab modules [12] letting us interactively define time-dependent 3D positions on image planes that directly correspond to vertices $\mathcal {V}^{PM}_{i}$ as well as select vertices $\mathcal {V}_{j}$ on the triangular mesh representing the valve. Each mesh vertex $\mathcal {V}_{j}$ is associated with an initial 3D position $\mathbf {p}_{j}(t), \; t = 0$ and moves according to the simulation for $t > 0$, whereas the positions $\mathbf {p}_{i}(t), \; t \ge 0$ for vertices $\mathcal {V}^{PM}_{i}$ are prescribed to model additional Dirichlet boundary conditions for the papillary muscle tips, see Fig. 2a. The position of the PM tips can be interpolated between states. In case the positions for $\mathcal {V}^{PM}_{i}$s are defined heuristically (see below), the positions are static.

The cord edges $\mathcal {E}^{CT}_{ij}$ can be differentiated in primary and secondary depending on the position of the leaflet vertex $\mathcal {V}_{j}$ the cord is connected to. Edges with leaflet vertices close to the orifice are labeled primary, edges on the belly of the leaflet are labeled secondary. For each such edge, we add an additional distance constraint $C^{CT}(\mathbf {p}_{i}(t), \mathbf {p}_{j}(t))$ to the simulation. The influence of constraint $C^{CT}$ in the simulation is weighted with parameter $k^{CT}$, which can assume different values in the two cases where the edge is compressed or stretched:

1 $$\begin{aligned} k^{CT} = {\left\{ \begin{array}{ll} 0, &{} |\mathbf {p}_{j}(t) - \mathbf {p}_{i}(t)| - l_{0} < 0 \; \text {(compression)},\\ 1, &{} |\mathbf {p}_{j}(t) - \mathbf {p}_{i}(t)| - l_{0} \ge 0 \; \text {(stretching)}. \end{array}\right. } \end{aligned}$$

The value $l_{0} = |\mathbf {p}_{j}(t) - \mathbf {p}_{i}(t)|$ represents the rest length of the cord at time $t > 0$ that corresponds to the closed state. This value is unknown and needs to be determined. We suggest setting the initial lengths $l_{0}$ to the sum of two absolute differences: One absolute difference determines the distance of the user defined leaflet vertex $\mathcal {V}_{j}$ to the AP. The other absolute difference determines the distance of the user defined leaflet vertex $\mathcal {V}_{j}$ to the corresponding $\mathcal {V}^{PM}_{i}$.

In the XPBD solver, constraints *C* are parameterized using a compliance parameter $\alpha$ [7, 16]. In the cord case, i.e., for $C^{CT}$, compliance can be set either to $\alpha ^{CT} = 0$ or it can be set to a compliance value corresponding to the inverse of the Young’s modulus [7] appropriate for chordae tendineae. The Young’s modulus of chordae tendineae can be found in [3, 11] and differs, e.g., for primary and secondary cords. Using the former parameterization $\alpha ^{CT} = 0$, the tendinous cords cannot be stretched longer than their initial lengths $l_{0}$. This implies that the distance of the leaflet vertex $\mathcal {V}_{j}$ to the corresponding papillary muscle tip vertex $\mathcal {V}^{PM}_{i}$ is limited to approximately $l_{0}$. Using this approach, no further assumptions on the biomechanical properties of the patient’s cords are required. The latter approach of using a measured value for compliance models a linearly elastic cord. It can be parameterized based on literature values for the chordae tendineae, but in most cases, patient-specific values are out of reach in clinical routine. In both cases and when not under load, the vertices $\mathcal {V}_{j}$ can move freely.

**Fig. 3:**
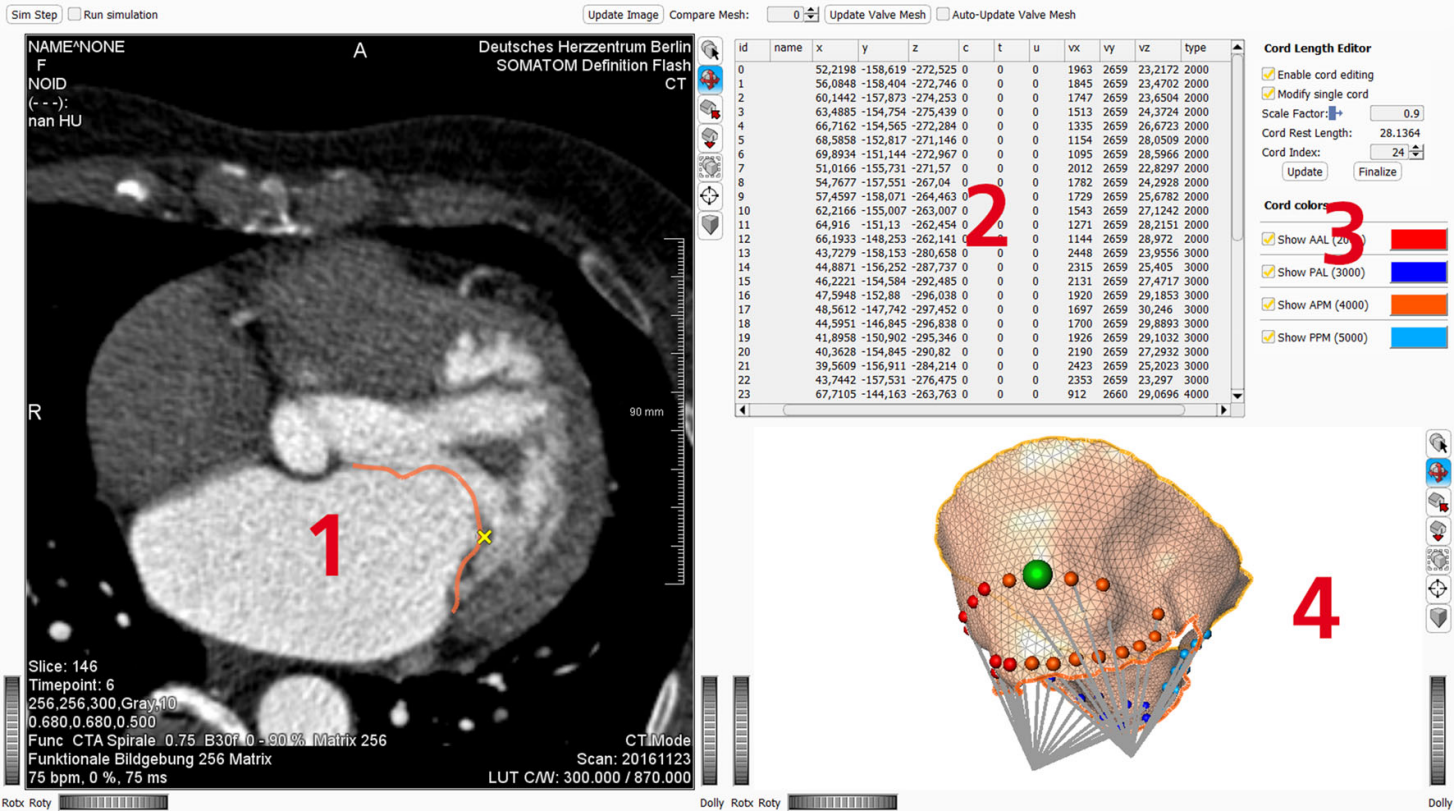
Overview of the interactive editor. The panel marked with **1** shows the image plane that belongs to the current selected cord end point marked as a yellow cross. The same cord end point is shown as a green sphere in panel **4** which depicts the 3D view of the open MV and the model cords. Panel **2** shows, in addition to coordinates and indices, the length of the cords. Panel **3** represents the interaction with the user. Here, the user can choose to scale individual cord lengths, or scale the lengths of one of the four groups of cords, or scale the lengths of all cords at once

Since the papillary muscles can sometimes not be reliably determined in the image data recorded in medical routine, a heuristic [16] can be used to initially define positions for $\mathcal {V}^{PM}_{i}$ (cf. Fig. 2b). The heuristic definition of $\mathcal {V}^{PM}_{i}$ depends on the set of manually defined vertices $\mathcal {V}_{j}$ that correspond to the primary and secondary cords. Using the position information of the leaflet vertices and the AP, we define the model cords. In the following, we assume just two papillary muscle tips $\mathcal {V}^{PM}_{\{0,1\}}$ which represent the connection of the leaflets to the posteromedial and anterolateral papillary muscles. The set of user-defined leaflet vertices $\mathcal {V}_{j}$ is manually divided into two subsets. About half of the vertices of the anterior and posterior leaflet (lighter colored vertices in Fig. 2b) are connected to one PM tip. The other half (darker colored vertices in Fig. 2b) is connected to the other tip. Light and dark colored spheres indicate the connectivity to one of the PM tips in Fig. 2b. Red and blue colored spheres distinguish between anterior and posterior leaflets. The centers of gravity $\mathbf {c}_{\{0,1\}}$ of both subsets of leaflet vertices are calculated and the distances $d_{\{0,1\}}$ of $\mathbf {c}_{\{0,1\}}$ to the AP are determined. The final tip positions for $\mathcal {V}^{PM}_{\{0,1\}}$ are calculated to be $\mathbf {p}_{i}(t) := \mathbf {c}_{i} \pm d_{i} \cdot \mathbf {n}^{AP}, \, i \in \{0,1\}$. The sign is chosen depending on the direction of $\mathbf {n}^{AP}$. A detailed example of the involved vertices, centers of gravity, and the links created can be found in Fig. 5b.

### Interactive editor

After the initial definition of the chordae tendineae, the interactive editor can be used to improve the approximation of the closed state valve. The method can be applied to MVs with normal or abnormal closing behavior.

**Fig. 4:**
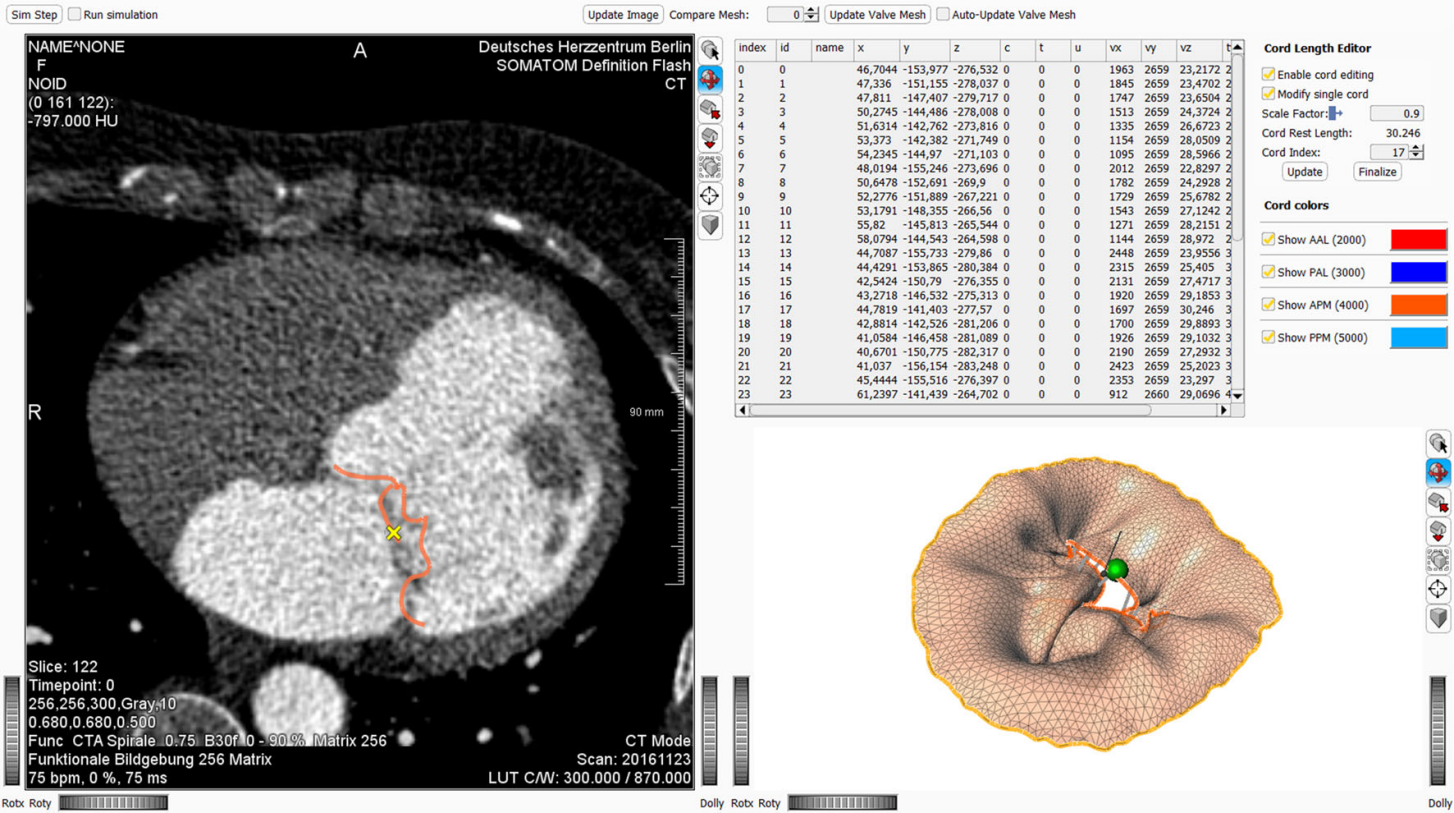
Interactive editor showing the closed valve. The selected cord end point is shown in yellow in panel 1 and in green in panel 4 (see Fig. 3 for panel numbering). The prolapsing valve is overlaid on the image data for visual comparison. By using the controls in the top right corner (panel 3), the cord lengths can be manipulated while the simulation is running. This way, changes can be evaluated immediately

As a first step, the closure of the valve can be simulated using the parameterization described above. The approximation can be evaluated by comparing cross sections of the valve mesh to the corresponding image planes. In case further tweaking of the cord model is necessary to improve the approximation, previous steps in the definition of the cord model can be revisited and modifications can be made to the leaflet end vertices $\mathcal {V}_{j}$ (regarding changes in number or initial positioning on the leaflet surface) or the PM tips $\mathcal {V}^{PM}_{i}$ (refinement of the manually or heuristically defined position), see previous section. Based on these definitions, the rest lengths of each individual cord can be modified by the user to define an optimized set of cords $\{\mathcal {E}^{CT}_{ij}\}$ for a MV data set. Our prototypical MeVisLab implementation provides an interactive editor (see Figs. 3, 4) where the user can choose to scale individual cord lengths or scale the lengths of one of the four groups of cords (anterior or posterior leaflet vertices connecting to either anterolateral or posteromedial papillary muscle tips) or scale the lengths of all cords at once. Technically, this updates the rest lengths $l_{0}$ of the distance constraints $C^{CT}$. The update can be performed while the simulation is being executed. The results are immediately visible to the user. In addition, the user can switch the force fields between closure and opening to see the updated dynamics using the new cord rest lengths. By changing between the states, the user can also get an intuitive idea how to change the lengths to create coaptation or prolapse in a certain area of the valve. To assess the achieved approximation, cross sections of the valve mesh can be continuously displayed superimposed on the image series. By selecting an individual leaflet end vertex $\mathcal {V}_{j}$, the image shown to the user can be switched to the corresponding plane. While the simulation is running, the effects of scaling a cord can be compared to the image data. By changing the rest length, the resulting movement of the end vertex with respect to the image plane can also be seen, e.g., if the end vertex just moves up or down in the same plane or tends to leave the current image plane shown to a plane above or below.

## Results

**Fig. 5:**
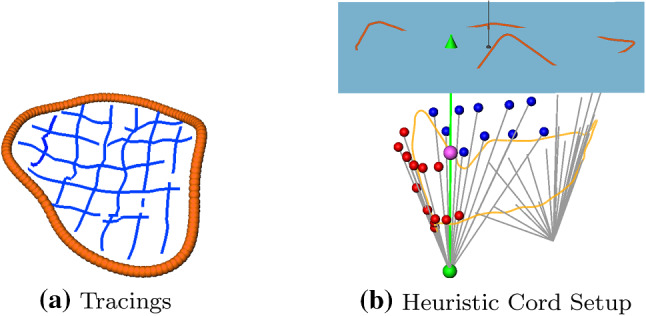
**a** Example of manually drawn tracings (shown in blue) of a closed valve shown in 3D. The tracings were defined in 2D on a limited number of image planes of the entire volume. The annulus contour is shown in dark orange. **b** Detail view of the cord setup in 3D. The annulus plane (AP) is shown as a blue rectangle, while the annulus center of gravity is depicted as a black sphere in its center. The AP normal is shown as a black line originating from the center. The annulus contour intersects the AP and is shown in dark orange if located above the AP. The orifice contour is shown in light orange. The manually placed leaflet vertices marking the cord end points are shown as red (anterior) and blue (posterior) spheres. The pink sphere marks the center of gravity of the chosen leaflet vertices, the green sphere shows the corresponding heuristically calculated papillary muscle tip. The green line is parallel to the AP normal and points to the AP. It touches the center of gravity of the chosen vertices (see construction in text). The gray lines depict the generated cords in open state and are shown for both PM tips

We used the interactive editor described above to improve the approximation of five pathological MVs exhibiting prolapse. The pathological cases were already used in [16] for qualitative evaluation of the closing behavior. The cases were named $B1{-}B5$. In [16], we were able to reproduce prolapse in the correct location in two of five cases using the heuristic. Using the editor, we attempted to reproduce a correct prolapse for the remaining cases. Furthermore, we examined the deviation of the simulation results to the closed valves and tried to improve the geometric approximation for all cases. The approximation quality in different simulation setups was assessed by comparing the distances of the simulated closed valve to manually drawn tracings of the closed valve seen in the image series (cf. Fig. 5a). The resulting deviations are listed in Table 1.

In cases *B*2–*B*5 prolapse was reconstructed in the right position and the approximation could be improved. In case *B*1 however, prolapse could not be reconstructed. The cord parameterization could be found in less than five minutes per case using the interaction described earlier.

## Discussion

**Fig. 6:**
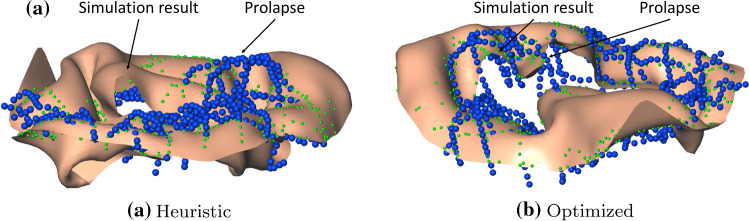
**a** Overlay of simulation result (brown), manual tracings (blue) and distance measurements (green, projection of manual tracings onto valve surface). The tracings can be used to assess the pathology as well as the simulation outcome. The case shown is B5 in which the pathology was predicted in a different location on the opposite leaflet using the heuristic parameterization. **b** By changing individual cords lengths, the result was vastly improved. The prolapse is now in the right location and the geometry shows good approximation

Using the interactive editor, we were able to reproduce prolapse in four of the five cases compared to two of five cases using just the heuristic. We were also able to improve the maximum, median as well as average deviation to the tracings in four of the five cases. The cases *B*3 and *B*4 reproduced the prolapse before and after cord length adjustment, but afterwards with better approximation. After cord length optimization, the cases *B*2 and *B*5 reproduced prolapse in the correct location with better overall approximation. The improvements that could be achieved using the editor are shown in more detail for case *B*5 in Fig. 6a, b. In Fig. 6a, the result using the heuristic is depicted. The simulation result for closing exhibits prolapse, but it is located at the wrong location. Using the editor, the result could be improved. The prolapse is located in the correct location (see Fig. 6b), and the tracings are more closely approximated. The overall deviations listed in Table 1 are comparable to other simulation approaches [2, 8], although we could only find values for normally closing MVs. To our knowledge, there are no descriptions of simulations results for MVs with abnormal closing behavior. The case *B*1 did not improve because of an overestimated leaflet size. The attempt to improve the approximation lead to bulging under the closed valve and in turn to larger deviations.

**Table 1:** Case-specific valve surface area (left column) and deviations to the expert annotations (right columns)

Case	Area ($\hbox {mm}^2$)	MinD (mm)	MedD (mm)	MaxD (mm)	AvgD (mm)	Prolapse Recon.
B1	3312	0.01	1.63	6.12	$1.86\pm 1.34$	No
B1 opt	3312	0.00	1.77	7.39	$2.06\pm 1.60$	No
B2	3531	0.00	2.05	8.23	$2.49\pm 1.88$	No
B2 opt	3531	0.00	1.66	7.94	$2.11\pm 1.70$	Yes
B3	2860	0.01	1.82	6.92	$2.12\pm 1.59$	Yes
B3 opt	2860	0.00	1.77	5.96	$1.93\pm 1.31$	Yes
B4	2312	0.02	1.57	6.68	$1.88\pm 1.40$	Yes
B4 opt	2312	0.01	1.74	4.86	$1.88\pm 1.13$	Yes
B5	3855	0.00	1.69	8.33	$2.20\pm 1.82$	No
B5 opt	3855	0.00	1.55	5.54	$1.77\pm 1.26$	Yes

These deviations are provided for the heuristically created CT or with individually scaled CT (the latter is referred to as **opt**). Cases B refer to pathological MVs. **MinD**, **MedD**, **MaxD** and **AvgD** refer to minimum, median, maximum and average deviation from the manually determined close valve contours. **Prolapse Recon.** indicates for both cases whether the prolapse was correctly reconstructed or not

Regarding the interaction itself, a suitable parameterization for the cord model could be found in four of the five cases. In one case where the parameterization was not possible, the error could be attributed to leaflet size. Exact initial reconstructions are crucial for successful simulations. The time needed to come up with a parameterization was acceptable at less than five minutes per case. Directly visible simulation results helped in that regard. Drawing tracings of the closed MV and comparing the cross sections of the valve mesh to the image data or to tracings on corresponding image planes posed no challenge in general, although out-of-plane movement was confusing at times when cross sections of the valve mesh appeared and disappeared. Inter- and intra-observer variability was not analyzed yet.

After successful model building, additional work steps are needed to define a set of virtual interventions (e.g., annulus ring implant [10], clipping, cord manipulation). The influence of the manipulation can directly be seen in the simulation, see [16] for more details.

## Conclusion

Our approach is suitable to create realistically parameterized mitral valve apparatus reconstructions for the simulation of normally and abnormally closing mitral valves. We did not find other approaches that are able to achieve comparable results. The parameterization using the editor is necessary because of uncertainties in the image data. In four of five cases, the reproduction of the pathology as well as the approximation quality could be improved. Together with tools defining virtual interventions, the resulting model can be used in decision support. In the future, we are going to implement an automatic placement of leaflet vertices and an inverse solver for an automatic determination of the cord rest length.
